# The complete chloroplast genome of *Primula tsiangii* W. W. Smith (Primulaceae): a karst endemic primrose in Southwest China

**DOI:** 10.1080/23802359.2019.1642160

**Published:** 2019-07-17

**Authors:** Sheng Chen, Xiaokai Yan, Gang Hao, Yuan Xu

**Affiliations:** a College of Life Sciences, South China Agricultural University, Guangzhou, China;; b Key Laboratory of Plant Resources Conservation and Sustainable Utilization, South China Botanical Garden, Chinese Academy of Sciences, Guangzhou, China

**Keywords:** *Primula tsiangii*, plastid genome, karst endemic plant, Illumina sequencing

## Abstract

*Primula tsiangii* W. W. Smith is a karst endemic species in Southwest China. Here, we report the complete chloroplast genome of *P. tsiangii*. The chloroplast (cp) genome was determined to be 153,281 bp and the GC contents was 37.2%. The sequence includes a large single copy (LSC) region of 84,524 bp, a small single copy (SSC) region of 17,357 bp, and two separated inverted regions of 25,700 bp each. It contains 127 unique genes, including 82 protein-coding genes, 37 tRNA genes, and 8 rRNA genes. This is the first report of cp genomes for karst endemic primrose, and will be useful for the genetic diversity of karst plants.

The subtropical mountains of Southwest China have been recognized as one of the world centers of plant diversity (Davis et al. 1995) and are also inferred as the center of origin of *Primula* L. (Hu 1994). High species endemism have been found in the karst areas of this region (Hao et al. 2015). Recently, several new species of *Primula* have been reported in the subtropical mountains (e.g. Xu et al. 2017). Most of them are endemic in the karst landform. *Primula tsiangii* W. W. Smith is one of the representative karst plant which only occurs on the limestone cliff. In this study, we report the complete chloroplast genome (cp genome) of *P. tsiangii* to provide new insight into the evolution of *Primula* and the genetic diversity of karst flora.

The fresh and healthy leaves of *P. tsiangii* were collected from Loushan Pass in Tongzi County, Zunyi City, Guizhou Province, China. The voucher specimens (voucher no.: Xu150008) was deposited at the herbarium of South China Botanical Garden (IBSC). The total genomic DNA was extracted following the CTAB method (Doyle and Doyle 1987). Then the genomic library (paired-end, PE = 150 bp) was sequenced on an Illumine Hiseq 4000 platform at Beijing Genomics Institute (Shenzhen, China). Totally 2 Gb sequence reads were obtained and used to assemble the cp genome after filtering and trimming the low-quality reads and adaptor sequences. The complete cp genome assembly was executed on A5-miseq pipeline (Coil et al. 2015), with manual adjustment and annotation using Geneious version 11.0.3 (Kearse et al. 2012). *Primula poissonii* (GenBank accession number: NC_024543) was used as reference plastid genome for assembling and annotation. The tRNA genes were annotated on ARAGORN (Laslett and Canback, 2004).

The complete cp genome of *P. tsiangii* (GenBank accession number: MN065496) was 153,281 bp in length, including a small single copy region (SSC) of 17357 bp, a large single copy region (LSC) of 84,524 bp and a pair of inverted repeats (IRs) of 25,700 bp. The genome harbors 127 unique genes, including 37 tRNA genes, eight rRNA genes, and 82 protein-coding genes. Among these genes, 11 genes have one intron and three genes (*clpP*, *rps*12, and *ycf*3) have two introns. The GC contents of this genome are 37.2%, while the GC contents of SSC, LSC, and IRs are 30.8%, 35.1%, and 42.8% respectively.

To identify the phylogenetic position of *P. tsiangii* in the *Primula*, a maximum likelihood (ML) tree was reconstructed by using RAxML (Stamatakis 2006) with 1000 bootstrap replicates. The matrix, used for phylogenetic analysis, includes 11 whole cp genome sequences of *Primula* species (10 of them download from GenBank) and was aligned in MAFFT (Katoh and Standley 2013). *Androsace laxa* C.M. Hu et Y.C. Yang and *Lysimachia coreana* Nakai were selected as outgroups. The phylogenetic tree indicates that *P. tsiangii* is closely related to *P. persimilis* G. Hao, C.M. Hu & Y. Xu (Figure 1). Both of them belong to sect. *Monocarpicae* Franch. ex Pax, forming a monophyletic clade with strong bootstrap support (Figure 1).

**Figure 1.**
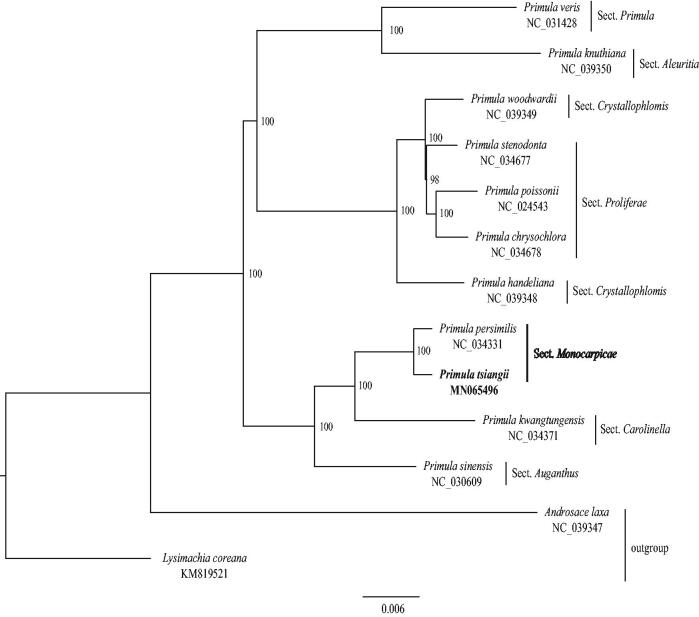
Maximum-likelihood tree based on 13 complete chloroplast genomes of Primulaceae. *Androsace laxa* and *Lysimachia coreana* were used as outgroups. Bootstrap support values are shown at the branches.
